# Biosorption of metribuzin pesticide by Cucumber (*Cucumis sativus*) peels-zinc oxide nanoparticles composite

**DOI:** 10.1038/s41598-022-09860-z

**Published:** 2022-04-07

**Authors:** Atta ul Haq, Muhammad Saeed, Majid Muneer, Muhammad Asghar Jamal, Tahir Maqbool, Tayyab Tahir

**Affiliations:** Department of Chemistry, Government College University, Faisalabad, 38000 Pakistan

**Keywords:** Environmental sciences, Natural hazards, Chemistry, Materials science, Nanoscience and technology

## Abstract

Herein, a biosorbent was prepared from cucumber peels modified with ZnO nanoparticles (CPZiONp-composite) for the biosorption of metribuzin. Characterization of the composite was accomplished using FTIR, SEM, EDX, surface area pore size analyzer and pH of point of zero charge (pH_pzc_). Biosorption study was executed in batch concerning the impact of pH, composite dose, contact time, initial metribuzin concentration and temperature. The biosorption depends on pH and maximum biosorption was acquired at pH 3.0. Surface chemistry of the composite was studied by determining the pH_pzc_ and was found to be 6.1. The biosorption nature was investigated using isotherms and was assessed that Freundlich isotherm is well suited for the fitting of the biosorption data owing to the highest R^2^. The maximum biosorption capacity of CPZiONp-composite was found to be 200 mg g^-1^. The biosorption data were fitted in to different kinetic models and the outcomes suggesting that pseudo second order is a satisfactory model to interpret the biosorption data owing to the highest R^2^. Thermodynamic parameters for instance entropy, enthalpy and Gibbs free energy were computed and revealed that biosorption of metribuzin onto CPZiONp-composite is spontaneous and exothermic process.

## Introduction

Food products such as vegetables and fruits are a principal part of a balanced diet for humans which are important sources of minerals, vitamins, dietary fiber, and phytochemicals^1^. Therefore, pesticides are used globally for the control and elimination of pests and to protect fruit and vegetable diseases^2^. However, with the advantages of the use of pesticides, they are still treated as toxic substances and cause environmental hazards, particularly when used improperly and randomly. The inappropriate use of pesticides may result in extensive discharge to the environment, that not only affects the human heath but also disturb the ecosystems^3^.

Metribuzin (MB) belong to triazinone herbicide (4-amino-6-tert-4, 5-dihydro-3-methylthio-1, 2, 4- triazin- 5-one) and usually utilized for the pre-emergence and post-emergence treatment of different grasses and broadleaved weeds in diverse crops for instance tomato, soya beans, barley, alfalfa, potato, maize and sugarcane. Metribuzin kills the target plant via coupling with the proteins of photosystem II complexes which thus restrains the process of photosynthesis^4^. High solubility of metribuzin in water (1050 mg L^-1^) and less sorption of metribuzin in soil make it risk not only for the surface but also hazard for water contamination^5–8^. Metribuzin is incorporated in the aqueous system via spray, leaching and flowing from treated soil, vapor drift and accidental discharge, and may lead to water contamination. It has been reported that metribuzin is very harmful to algae and fresh water macrophytes^9^. Metribuzin may also give rise to physiological disorders in fauna via disrupting the endocrine system. Hence, it has been admitted in different countries in regulated endocrine disruptors. Moreover, residues of metribuzin may cause disturbing of crop rotation system via influencing the establishment of successive crop^10,11^. Therefore, United States Environmental Protection Agency recommenced a particular concentration (175 µg L^-1^) of metribuzin in drinking water^12^.

In the recent past, numerous procedures have been anticipated to get rid from pesticides in water for instance ultrasound combined with photo-Fenton treatment^13^, electrodialysis membranes^14^, Photocatalytic degradation^15^, advanced oxidation processes^16^, ozonation^17^, and aerobic degradation^18^ but however, these methods have some serious limitations and demerits such as ineffectiveness, high cost, and are not environment-friendly due to which these procedures have not been extensively applied for the removal of pesticides^19^. Therefore, researchers are giving more attention to investigate methods which are economical, effective and eco-friendly. At the present time, biosorption is one of the most promising and alternative method which have gotten much more attention owing to economical, effectiveness and free of secondary pollution^20^. Biosorption is a process in which contaminant binds from water solution onto functional groups of the surface of biomass. Previously, an abundance of biomasses was investigated for treatment of wastewater containing pesticides for instance neem seeds powder^21^, pumpkin seeds shell powder^22^, Pea peels^23^, fungus Pleurotus mutilus^24^, cork species^25^, bark^26^, straw^27^, coal fly ash^28^ and activated carbon^29^.

Biosorbents have soft nature and have the propensity of agglomeration or formation of gel in water as a result the available active sites of the biomasses decreased for the biding of pollutants. Furthermore, physical support of the biosorbents is also essential to increase the accessibility of pollutants toward the sorption sites^30^. Therefore, it has been acknowledged to enhance the binding ability of biosorbents by further modification. For this purpose, many efforts have been accomplished to improve the sorption capability of biosorbents^31–38^. However, biosorbents impregnated with metal oxides have gained substantially more attention owing to distinctive characteristics of the metal oxides such as large surface area and small size^39^. Furthermore, metal oxides enhanced the stiffness and hardiness of polymeric materials and thus increase the durability of polymeric materials^40^.

While keeping the aforesaid reason in mind, an attempt was performed to synthesize cucumber peels-zinc oxide nanoparticles composite to remove metribuzin from aqueous medium. The biosorption of metribuzin was carried out in respect of the impact of the pH, composite dose, temperature, contact time and initial metribuzin concentration in order to obtain optimized experimental conditions for maximum biosorption of metribuzin from aqueous medium.

## Materials and methods

### Chemicals and reagents

The chemicals and reagents used in the recent research paper were of analytical grade. All the chemicals and reagents were purchased from the Merck^®^ and Sigma-Aldrich^®^. In the current research work zinc oxide (CAS No. 1314–13-2), phosphoric acid (CAS No. 7664–38-2), nitric acid (CAS No. 7697–37-2), acetic acid (CAS No. 64–19-7), boric acid (CAS No. 10043–35-3), sodium hydroxide (CAS No. 1310–73-2) and metribuzin (CAS No. 21087–64-9) pesticide were employed with no additional purification.

### Instruments

In this study analytical balance (PA413 OHAUS Corporation, USA) was utilized to weigh solids while solid materials were ground with the help of electrical grinder (Frtsch-Pulverisette 2 of Japan). Sonicator (Model: DSA100-SK1-2.8L) was used for sonication during preparation of composite of cucumber peels and zinc oxide nanoparticles. The orbital shaker was used to shake the composite and metribuzin solution for different interval of times. To measure the pH of solution, pH meter (WTW-Inolab pH7110) was used while electrical oven (Memmert Celsius 2005) was used to dry the composite after preparation. To determine the concentration of metribuzin before and after biosorption studies UV/Vis spectrophotometer was used. The morphological study of the composite was performed using scanning electron microscope (SEM-Model-JSM-5910, Japan JEOL) while elemental analysis of the composite was carried out with the aid of Energy Dispersive X-ray (EDX-INCA 200 Oxford Instruments UK). The functional groups of raw material and composite were studied with the help of Fourier Transform Infrared spectrometer (Bruker ALPHA) and surface analysis of composite was studied by Surface Area Pore Size Analyzer (Model NOVA2200e, Quantachrome, USA).

### Standard solution of metribuzin

Firstly, a standard solution of metribuzin of 1000 mg L^-1^ concentration was prepared by dissolving 0.142 g of metribuzin (70%) in distilled water in a beaker. After complete dissolution of metribuzin in beaker the solution was transferred in 100 mL volumetric flask. The solution was then diluted with distilled water up to mark. Then dilution formula was used to prepare working standards in 100 mL volumetric flasks from this stock solution for further research work.

### Collection of cucumber peels

All methods were performed in accordance with the relevant guidelines and regulations. The cucumber is a vegetable which people eat as a salad. The peels of the cucumber are a waste and the people throw them. It is collected as waste material from the market and utilized as biosorbent. Cucumber peels were obtained in large quantity from hotels around the Government College University Faisalabad, Pakistan. These peels were washed with distilled water numerous times and were put in sunlight for two weeks to dry. The dried material was then ground employing an electrical grinder. The powder of the cucumber peels was obtained and passed through a domestic sieve to separate any larger particles. Then cucumber peels powder was stored in airtight bottle for preparation of nanocomposite^41^.

### Preparation of CPZiONp-composite

An accurately weighed amount (approximately 0.75 g) of ZnO was dissolved in a mixture of 100 mL of CH_3_COOH (1%) and 10 mL of HNO_3_ (65%) in a conical flask. Then 1.0 g of cucumber peels powder was added into the flask and sonicated for half hour. When sonication was completed then NaOH (1.0 M) was added drop by drop until pH of the mixture become 10. The mixture was kept in water bath at 60 °C for 3 h. Flask’s content was filtered and the material was washed repeatedly with distilled water. After completion of washing of the material then it was dried at 50 °C for about 1 h in an oven. The dried material was then ground and stored for the biosorption of metribuzin in aqueous medium^42^.

### Determination of pH of point of zero charge (pH_ZPC_)

The salt addition procedure was followed for the determination of pH_ZPC_ of CPZiONp-composite^43^. In this method an accurately weighed amount of CPZiONp-composite (0.1 g) was taken in a series of beakers containing 95 mL of 0.01 M sodium chloride. The pH of each beaker’s content was adjusted with 1.0 N hydrochloric acid and sodium hydroxide of each in the range of 2 to 10. The beaker’s contents were then kept for 6 h in a shaker bath at room temperature. After 6 h of equilibration the pH of each beaker’s content was determined again and plotted the difference, ΔpH (final pH – initial pH) versus initial pH. The pH_ZPC_ was evaluated at the intersection of initial pH with ΔpH in the plot.

### Biosorption experiment

Biosorption of metribuzin onto CPZiONp-composite was executed in batch mode by transferring 10 mL metribuzin in a concentration of 33–155 mg L^-1^ and added CPZiONp-composite in the range of 0.01–0.09 g in conical flasks. The initial solution pH was adjusted with Britton–Robinson buffer solution between 3 and 12 by adding 5 mL of each and the mixture was agitated in orbital shaker for 10–90 min at a temperature varying from 30 to 80 °C. The mixture was filtered after completion of fixed stirring times and concentration of metribuzin was determined at maximum wavelength (290 nm) by the help of UV/Vis spectrophotometer.

Percentage biosorption and biosorption capacity of metribuzin onto CPZiONp-composite were determined using the following formulae:

1 $$ Biosorption \left( \% \right) = \left[ {\frac{{C_{i} - C_{e} }}{{C_{i} }}} \right] \times 100 $$

2 $$ Biosorption\, capacity \left( {Q_{e} } \right) = \left[ {\frac{{C_{i} - C_{e} }}{W}} \right]V $$

Here C_i_ signifies the initial concentration (mg L^-1^) and C_e_ implies equilibrium concentration (mg L^-1^), W symbolizes dose of biosorbent (g) and V indicates volume of solution in milliliter.

### Isotherms study

In the isotherms study, solutions of the metribuzin were prepared in the concentrations range of 33–155 mg L^-1^, buffered to pH 3.0 with Britton–Robinson buffer solution and added 0.05 g of CPZiONp-composite in the conical flasks. The mixture was agitated for 90 min in orbital shaker at a temperature of 30 °C. The mixture was filtered and concentration of metribuzin was determined and analyzed according to the Freundlich, Langmuir, Temkin and D-R isotherms.

The biosorption isotherm exemplifies the relationship between biosorbent and biosorbate at a fixed temperature. As a consequence, biosorption isotherms are essential for optimization of the use of biosorbents. It is probable to demonstrate the equilibrium biosorption isotherm by plotting the amount of biosorbate in the solid phase versus the amount of biosorbate in the liquid phase^44^. Herein, biosorption data of metribuzin was examined via employing Freundlich, Langmuir, Temkin and D-R isotherms.

The Freundlich isotherm is one of the preliminary recognized empirical formula that exhibits invariability upon exponential distribution of biosorptive sites on a heterogeneous surface. This isotherm is usually used for multilayer biosorption on heterogeneous surfaces. It is explained from the following expression:

3 $$ ln\left( {Q_{e} } \right) = ln\left( {K_{F} } \right) + \frac{1}{n}ln\left( {C_{e} } \right) $$

Here Q_e_ (mg g^-1^) signifies the amount of metribuzin biosorbed onto CPZiONp-composite, K_F_ (mg g^-1^) exhibits the biosorption capacity, C_e_ (µg mL^-1^) means the concentration of metribuzin at equilibrium and 1/n indicates the biosorption intensity. It has been recognized that values of 1/n give information regarding the types of isotherm. It is recognized that the isotherm should be irreversible in case of (1/n < 0), desirable in case of (1/n > 0), or undesirable in case of (1/n > 1)^45^.

The Langmuir isotherm demonstrates that biosorption takes place in a monolayer on homogeneous surface composed of limited number of available sites which are energetically equal with each other. Biosorption energy remains unchanged and does not depend on the degree of filling of biosorptive active sites^46^. The empirical formula of this isotherm may be given as:

4 $$ \frac{{C_{e} }}{{Q_{e} }} = \frac{1}{{Q_{max} K_{L} }} + \frac{{C_{e} }}{{Q_{max} }} $$

Here Q_e_ (mg g^-1^) signifies the amount of metribuzin biosorbed onto CPZiONp-composite, Q_max_ (mg g^-1^) reveals the maximum biosorption capacity, C_e_ (µg mL^-1^) denotes the concentration of metribuzin at equilibrium and K_L_ (L mg^-1^) is associated with biosorption energy.

One of the most important characteristics of the Langmuir isotherm may be represented with regard to a dimensionless constant known as separation factor (R_L_). The magnitudes of R_L_ may be calculated by employing the following formula:

5 $$ R_{L} = \frac{1}{{1 + K_{L} C_{0} }} $$

Here K_L_ (L mg^-1^) denotes Langmuir constant and C_o_ (µg mL^-1^) signifies the initial concentration of metribuzin. The shape of isotherm may be inferred by obtaining R_L_ value. The biosorption may be favorable in case of (0 < R_L_ < 1), unfavorable in case of (R_L_ > 1), linear in case of (R_L_ = 1) or irreversible in case of (R_L_ = 0)^47^.

The Temkin isotherm considers that heat of biosorption fall with augmentation of dose of biosorbent in a linear mode. This isotherm consists of a parameter which designates obviously the interaction between biosorbate and biosorbent^48^. The following equation expresses this isotherm:

6 $$ Q_{e} = Blna_{T} + BlnC_{e} $$

7 $$ B = \frac{RT}{{b_{T} }} $$

Here a_T_ expresses the equilibrium bond constant, R signifies the gas constant, T means temperature while B and b_T_ are constants and associated with heat of biosorption.

The Dubinin–Radushkevich (D‒R) may help to check the kind of biosorption process that whether it is chemical or physical. This isotherm may be represented by the following equation:

8 $$ lnQ_{e} = lnQ_{m} - \beta \varepsilon^{2} $$

Here Q_m_ (mg g^-1^) serves the maximum biosorption capacity, β (mol^2^ J^-2^) denotes the activity coefficient, Q_e_ (mg g^-1^) expresses biosorption capacity while ε is known as the Polanyi potential. The value of Polanyi potential may be computed by employing the following equation:

9 $$ \varepsilon = RTln\left[ {1 + \frac{1}{{C_{e} }}} \right] $$

The constant β of the D‒R isotherm gives information concerning the average free energy E (kJmol^-1^) of biosorption process. It states that the energy expended during the shifting of one mole of biosorbate molecules from the solution to the surface of biosorbent. The average free energy may be computed from the following relationship.

10 $$ E = \frac{1}{{\sqrt {2\beta } }} $$

The value of E offers details regarding the nature of biosorption process that whether it occurs through physically or chemically. If E < 8 kJmol^-1^ then the biosorption process happens via physiosorption and if the E is within 8 to16 kJmol^-1^ then the biosorption process occurs through chemisorption^49^.

### Kinetic study

In this study, a fixed initial concentration of metribuzin of 33 mg L^-1^ was transferred in a conical flask, buffered to pH 3.0 with Britton–Robinson buffer solution and added 0.05 g of CPZiONp-composite. The mixture was agitated in the range of 10 to 90 min in orbital shaker at a temperature of 30 °C. The mixture was filtered and concentration of metribuzin was determined and analyzed according to the kinetic models of pseudo first order, pseudo second order, Elovich and Intraparticle diffusion.

The kinetics of the biosorption furnishes understandings about the dynamics of pesticide biosorption. Furthermore, it also assists to explore the controlling mechanism like chemical reaction, diffusion control or mass transfer of the biosorption process. The biosorption kinetics narrates about the amount of biosorbate biosorbed on the surface of biosorbent, which resultantly controls the time spends by biosorbate at the interface between biosorbent and solution^50^. In an effort to probe the control mechanism of the metribuzin biosorption onto CPZiONp-composite, the biosorption data was examined by utilizing pseudo first order, pseudo second order, Elovich and Intraparticle diffusion model.

For the first-time Lagergren presented the pseudo first order which can be employed for the determination of rate constant depends on the biosorption capacity. This model assumes that biosorption capacity changes as rate of change of the removal of biosorbate take place with respect to time of contact^51^. This model may be displayed in subsequent relationship:

11 $$ log\left( {Q_{e} - Q_{t} } \right) = logQ_{e} - \frac{{k_{1} \left( t \right)}}{2.303} $$

Here k_1_ (min^-1^) stands for rate constant of pseudo first order kinetic model, Q_e_ (mg g^-1^) signifies biosorption capacity at equilibrium and Q_t_ (mg g^-1^) means biosorption capacity at a time t.

The Ho and Mckay suggested pseudo second order which depending on the prediction of biosorption performance over the complete time required for biosorption. Moreover, this model assumes chemisorption mechanism of the biosorption process which takes place via valance forces from electron-sharing between pesticide and the functional groups on biosorbent^52^. Mathematical representation of this model is given below:

12 $$ \frac{t}{{Q_{t} }} = \frac{1}{{k_{2} Q_{e}^{2} }} + \frac{t}{{Q_{e} }} $$

Here k_2_ (gmg^-1^ min^-1^) symbolizes rate constant of pseudo second order kinetic model, Q_e_ (mg g^-1^) signifies biosorption capacity at equilibrium and Q_t_ (mg g^-1^) means biosorption capacity at a time t.

The Elovich model explains that biosorption occurs in the form of chemisorption on heterogeneous surfaces of the biosorbents^53^. The mathematical representation of the Elovich model is given in subsequent relationship:

13 $$ Q_{t} = \frac{1}{\beta }ln\left( {\alpha \beta } \right) + \frac{1}{\beta }ln\left( t \right) $$

Here α (mgg^-1^ min^-1^) corresponds to initial rate of biosorption and β (g mg^-1^) corresponds to degree of surface coverage.

The Morris and Weber proposed the Intraparticle diffusion model which is generally employed for the identification of diffusion mechanism of biosorption process. During the biosorption process different steps occur for instance transfer of biosorbate from the bulk solution to the external surface around the biosorbent, transfer of the biosorbate from the external surface of biosorbent to the interior sites and finally transfer of biosorbate from the interior sites to the interior surface of the biosorbent pores. Furthermore, Morris and Weber stated that if the plot of Q_t_ against t^1/2^ yields a straight line with zero intercept in that case intraparticle diffusion would be the rate determining step^54^. Mathematically, this model may be represented in subsequent relationship:

14 $$ Q_{t} = K_{id} \left( {\sqrt t } \right) + C $$

Here K_id_ (gmg^-1^ min^-1/2^) denotes rate constant of intraparticle diffusion and C (mg g^-1^) corresponds to thickness of the boundary layer.

#### Thermodynamic study

In this study, a fixed initial concentration of metribuzin of 33 mg L^-1^ was transferred in a conical flask, buffered to pH 3.0 with Britton–Robinson buffer solution and added 0.05 g of CPZiONp-composite. The mixture was agitated for 90 min in orbital shaker at temperature ranging from 30 to 80 °C. The mixture was filtered and concentration of metribuzin was determined and thermodynamic parameters such enthalpy, free energy and entropy were calculated applying Van't Hoff equation.

The nature and mechanism of the biosorption process may be studied by evaluating thermodynamic parameters. Thermodynamic parameters for instance standard free energy change (ΔG°), standard enthalpy change (ΔH°) and standard entropy change (ΔS°) indicate spontaneity, energy of interaction between biosorbate and biosorbent, and disorder of the system respectively^55^. These thermodynamic parameters were computed from the subsequent equations:

15 $$ K_{d} = \frac{{Q_{e} }}{{C_{e} }} $$

16 $$ \Delta G^\circ = - RTlnK_{d} $$

17 $$ lnK_{d} = - \frac{\Delta H^\circ }{{RT}} + \frac{\Delta S^\circ }{R} $$

Here K_d_ expresses thermodynamic equilibrium constant, Qe (mg g^-1^) represents biosorption capacity, C_e_ (µg mL^-1^) designates concentration of metribuzin at equilibrium, T (K) implies temperature, R stands for gas constant and has value of 8.314 JK^-1^ mol^-1^.

## Results and discussion

### Characterization of CPZiONp-composite

The raw material and prepared CPZiONp-composite were characterized before and after biosorption study using different techniques namely FTIR, SEM, EDX, surface area pore size analyzer and pH of point of zero charge.

#### FTIR analysis

**Figure 1:**
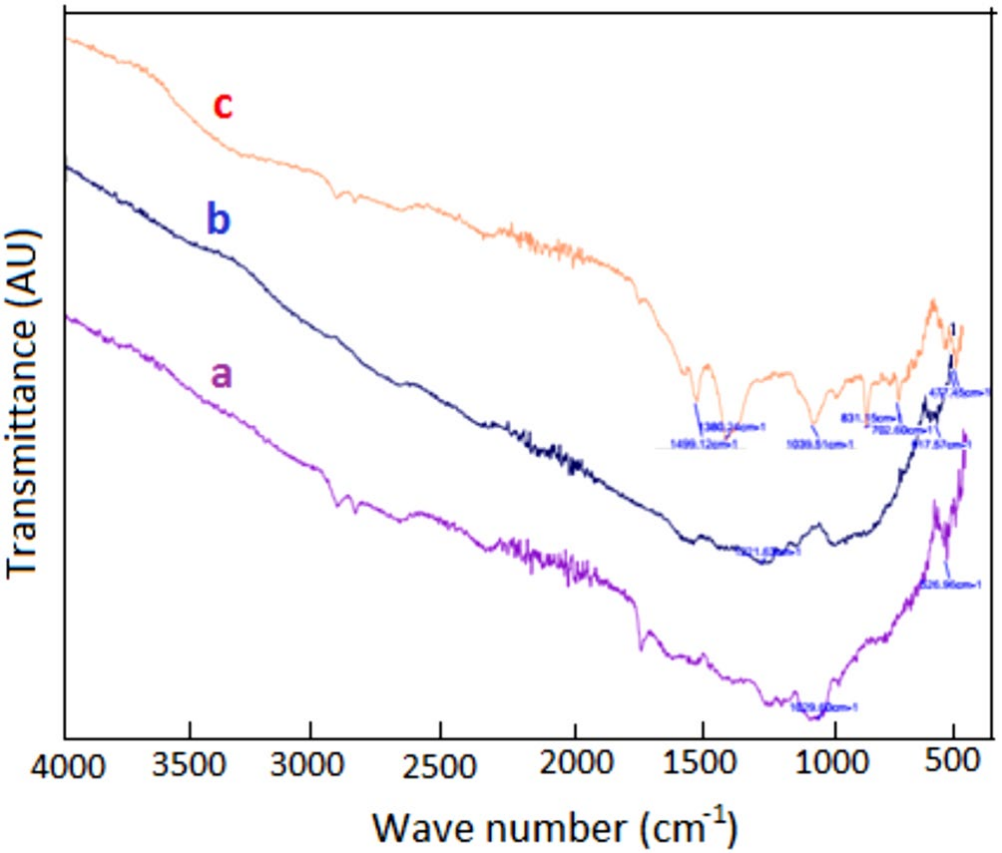
(**a**) FTIR spectra of material cucumber peel (**b**) CPZiONp-composite before biosorption (c) CPZiONp-composite after biosorption.

FTIR analysis of the CPZiONp-composite was accomplished for identification of functional groups and to examine the changes of functional groups in the course of biosorption^56^. It can be depicted from Fig. 1 that there are different peaks which may be assigned to certain functional groups regarding the wavenumber of each peak according to the literature. Figure 1a exhibits that cucumber peel has various peaks at different wavenumbers such as 2900, 1750, and 1029 cm^-1^ that correspond to C–H, C=O and C–O–C stretching vibration respectively. After impregnation of ZnO onto cucumber peels a peak between 500 and 400 cm^-1^ was appeared as illustrated in Fig. 1b which was allocated to O–Zn–O group vibration^57^. Furthermore, some new peaks were appeared after biosorption of metribuzin on CPZiONp-composite as depicted Fig. 1c such as 1499, 1380, and 1039 cm^-1^ which were allotted to C=N conjugated, C–N stretching and N=C–S stretching of metribuzin respectively. The appearance of additional peaks confirmed the biosorption of metribuzin onto CPZiONp-composite.

#### SEM analysis

**Figure 2:**
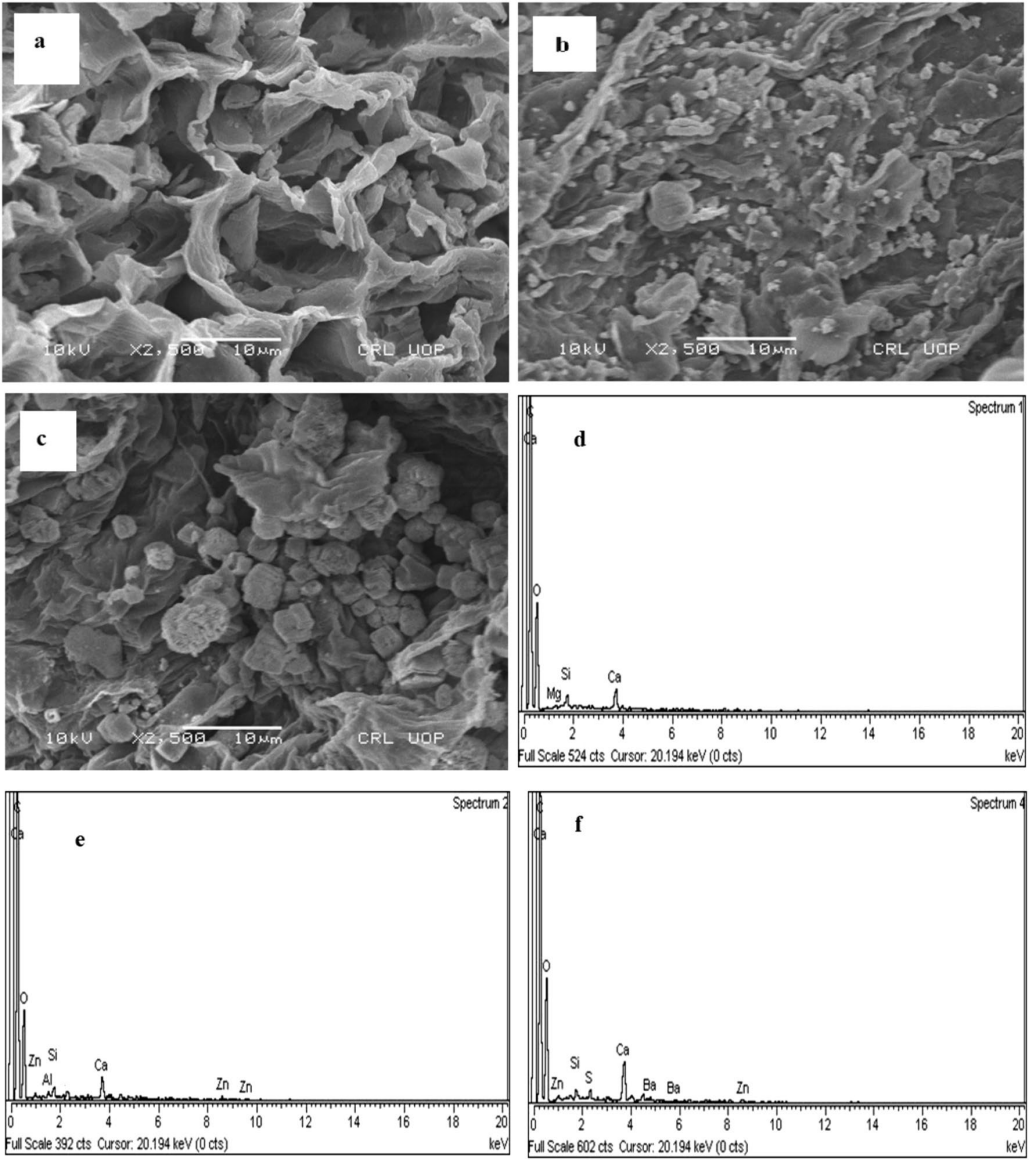
(**a**) SEM spectrum of cucumber peels (**b**) SEM spectrum of CPZiONp-composite (**c**) SEM spectrum of CPZiONp-composite after biosorption (**d**) EDX pattern of cucumber peels (**e**) EDX pattern of CPZiONp-composite (**f**) EDX pattern of CPZiONp-composite after biosorption.

The SEM analysis was executed for the identification and spotting of metribuzin molecules on the surface of CPZiONp-composite. There are several pores and cavities on the surface cucumber peels as depicted from Fig. 2a. However, after immobilization of ZnO onto cucumber peels the cavities become filled to some extent and the surface appeared smooth as shown in Fig. 2b. It may be depicted from Fig. 2c that numerous additional particles were shown after biosorption of metribuzin which designated the biosorption of metribuzin onto CPZiONp-composite.

#### EDX analysis

The changes in elemental composition of the cucumber peels and CPZiONp-composite was analyzed by energy dispersive x-rays (EDX) before and after biosorption of metribuzin. It may be depicted from Fig. 2d that EDX pattern of cucumber peels consists of C, Ca, O, Mg and Si. The presence of Zn element on CPZiONp-composite verified that ZnO was successfully immobilized on cucumber peels as illustrated in Fig. 2e. After biosorption study, the EDX pattern composed of S element as illustrated in Fig. 2f which suggests the biosorption of metribuzin onto CPZiONp-composite because metribuzin molecule contained sulfur atom.

#### Surface area analysis

It is common ground that surface area affects the biosorption capability of biosorbents. Hence, the surface area of cucumber peels and CPZiONp-composite was determined by N_2_ sorption isotherm. The result of surface area analysis is given in Table 1 which indicates that surface area of cucumber peels was increased after impregnation of ZnO onto cucumber peels. However, a reduction in surface area of CPZiONp-composite was occurred after biosorption of metribuzin which implies the biosorption of metribuzin onto CPZiONp-composite.

**Table 1:** Surface area analysis of cucumber peels and CPZiONp-composite.

Parameter	Cucumber peels	Before biosorption of metribuzin	After biosorption of metribuzin
Surface area (m^2^ g^-1^)	5.063	12.012	8.628
Pore volume (mL g^-1^)	15.078	15.002	15.067
Pore radius (Ǻ)	0.000	0.003	0.001

#### The pH of point of zero charge (pHpzc)

**Figure 3:**
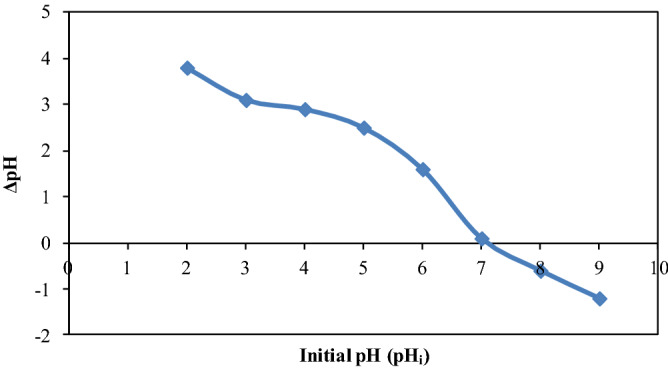
The pH of point of zero charge of CPZiONp-composite.

Charges on surface biosorbent play an essential contribution in biosorption and can be measured by determining pHpzc of biosorbent^58^. It states that the pH at which positive charges on biosorbent surface is numerically equal to the negative charges. It means that biosorbent surface has net zero charge at pHpzc. It is generally acknowledged that surface of the biosorbent has positive charges if pH < pHpzc while negative charges if pH > pHpzc^59^. Therefore, biosorption of cation is favored at a pH greater than pHpzc whereas biosorption of anion favored at a pH less than pHpzc^60^. It may be depicted from Fig. 3 that pHpzc of CPZiONp-composite is 7.0 which indicates that surface of CPZiONp-composite is positive charges < pH 7.0 and negative charges > pH 7.0. Therefore, for pH > 7.0, the biosorption of metribuzin is not favorable owing to repulsive electrostatic interaction between metribuzin molecules and negative charges of the surface. The maximum biosorption of metribuzin occurs at pH < 7.0 when the biosorbent surface is positively charged^61^. Hence, in the present study metribuzin was largely biosorbed at low pH due to strong electrostatic interaction between metribuzin molecules and CPZiONp-composite.

### Effect of pH

**Figure 4:**
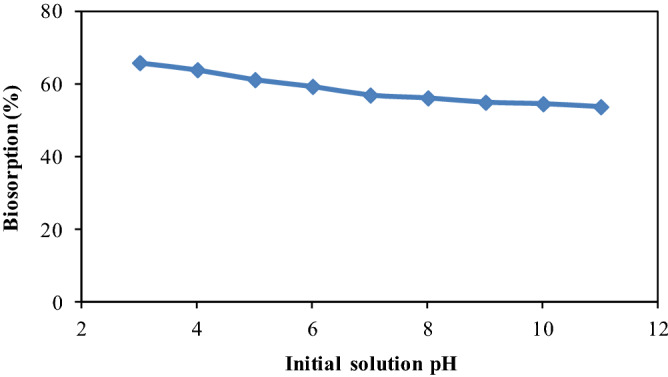
Effect of pH on biosorption of metribuzin.

There is widespread recognition that solution pH significantly influences the active sites and interaction between biosorbate and biosorbent^62^. Therefore, the biosorption of metribuzin was studied under the impact of pH of solution ranging from 3 to 11 with 33.33 µg mL^-1^ initial concentration of metribuzin. It may be depicted from Fig. 4 that biosorption of metribuzin gradually decreases with augmentation in pH and maximum biosorption was attained at low pH of solution. The pH_ZPC_ of CPZiONp-composite was 7.0 which suggested that at pH < pH_ZPC_, the surface of CPZiONp-composite was likely positive charge thereby increasing the biosorption of anionic metribuzin molecules due to electrostatic interaction. However, at pH > pH_ZPC_, the surface of CPZiONp-composite acquired increasing negative charge, inhibiting the biosorption of anionic metribuzin molecules^63^. Therefore, biosorption of metribuzin was constantly deceased with increase in pH and pH 3.0 then selected for further study.

### Effect of CPZiONp-composite dose

**Figure 5:**
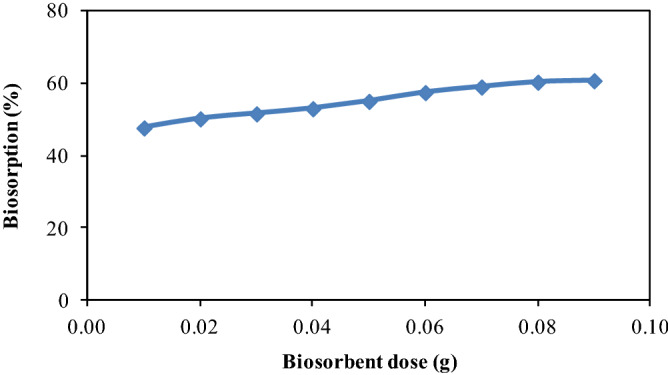
Effect of biosorbent dose on biosorption of metribuzin.

The ability of biosorbent to sequester biosorbate depends on biosorbent dose at a specified initial concentration of biosorbate^64^. Thereby, the influence of dose of CPZiONp-composite was examined by changing the dose of CPZiONp-composite between 0.01 and 0.09 g with 33.3 µg mL^-1^ of initial concentration of metribuzin. As it is evident from Fig. 5 that biosorption of metribuzin was enhanced gradually from 47 to 60 (%) with each increment in dose of CPZiONp-composite. Obviously, an increase in dose of CPZiONp-composite results to augmentation of active sites and enough surface area. Therefore, biosorption of metribuzin was enhanced with augmentation in dose of CPZiONp-composite till 0.08 g but no appreciable change in biosorption was observed after 0.08 g may be due overlapping or spatial inhibition of active sites in the presence of excess dose of biosorbent.

### Effect of contact time

**Figure 6:**
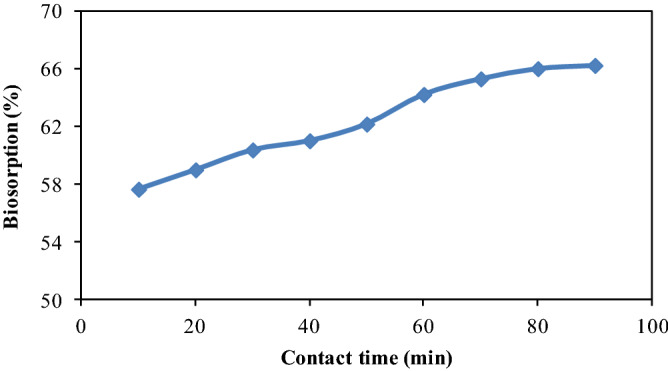
Effect of contact time on biosorption of metribuzin.

The capability of biosorbent to remove contaminants relies on the time span between biosorbent and biosorbate. Hence, CPZiONp-composite was stirred from 10 to 90 min in order to assess the equilibrium time for biosorption. As noticed in Fig. 6, the percentage biosorption of metribuzin was enhanced with rise in contact time and the percentage biosorption was augmented steadily up to 80 min. The biosorption of metribuzin was fairly fast in the initial stage of biosorption process but become slow down after 60 min of contact. However, the biosorption process was reached to equilibrium at 80 min and then no significant variation was noticed in the biosorption of metribuzin. Thus, further biosorption experiments were performed at 80 min of contact time.

### Effect of initial concentration of metribuzin

**Figure 7:**
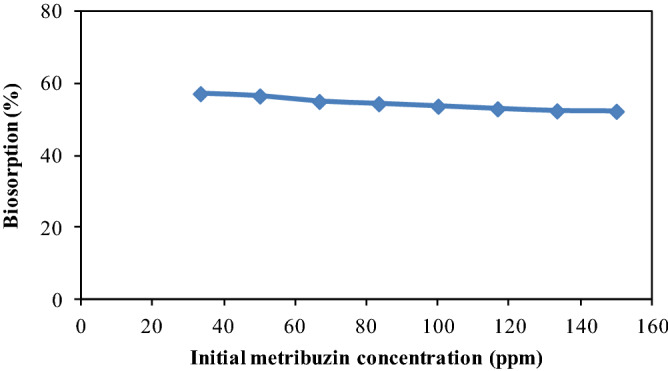
Effect of initial concentration of metribuzin on biosorption of metribuzin.

It has generally recognized that biosorption ability has greatly affected by initial concentration. For this reason, the impact of primary concentration of metribuzin on biosorption process was scrutinized at different initial concentration within the range of 33 to 155 µg mL^-1^ at 80 min of contact time and pH 3. As illustrated in Fig. 7 that biosorption of metribuzin onto CPZiONp-composite was higher at low initial concentration. However, the biosorption was declined constantly with augment in initial concentration of metribuzin. It can be explained that the number of metribuzin molecules at the beginning of biosorption was limited than the available site of CPZiONp-composite due to which maximum biosorption was observed at low initial concentration. Contrastingly, the number of metribuzin molecules at higher initial concentration was increased in contrast to available sites on CPZiONp-composite as a result of which biosorption of metribuzin were declined continuously^65^.

### Effect of temperature

**Figure 8:**
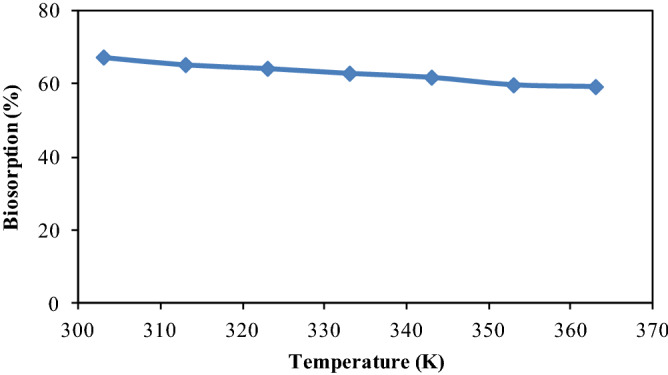
Effect of temperature on biosorption of metribuzin.

There is recognition that temperature significantly affects the biosorption process. For this reason, the biosorption of metribuzin onto CPZiONp-composite was studied at various temperatures within the range of 303–363 K at 80 min of contact time and pH 3. As shown in Fig. 8 that biosorption of metribuzin onto CPZiONp-composite was diminished steadily with rise in temperature. The lowering of biosorption efficiency with rise in temperature would be ascribed to the reduction in available biosorptive sites on the surface of CPZiONp-composite or the forces of attraction between metribuzin molecules and CPZiONp-composite^66^. As the temperature was increased the thickness of the boundary layer became small due to which metribuzin molecules easily got away from surface of CPZiONp-composite to the solution phase^67^. Consequently, biosorption of metribuzin onto CPZiONp-composite was decreased continuously with rise in temperature.

### Isotherm studies

All coefficients of each isotherm were computed using slope and intercept of linear plots of isotherms and magnitudes of the coefficients are presented in Table 2. The magnitudes of isotherm constants and correlation coefficients provides information about the fitness of the biosorption isotherms applied for the present study. Based on the value of R^2^ Freundlich isotherm generates a high compatibility to the experimental data than Langmuir, Temkin and D‒R owing to higher R^2^ value for the biosorption of metribuzin onto CPZiONp-composite. The Freundlich isotherm parameter 1/n suggests that biosorption of metribuzin onto CPZiONp-composite is desirable because its value is within the range of 0 < 1/n < 1. The parameters of Langmuir isotherm Q_e_ and K_L_ were found to be as 200 mg g^-1^ and 0.0041 (g mg^-1^ min^-1^). The magnitudes of R_L_ is ranged in between 0.6 to 0.8 which implies that biosorption of metribuzin onto CPZiONp-composite is favorable process. The values of correlation coefficients of Temkin and D-R isotherms indicates that Temkin D-R isotherms are not fit of equilibrium data as compared with the Freundlich and Langmuir isotherm models, respectively. The maximum biosorption capacity of CPZiONp-composite was computed from the Langmuir isotherm and was found to be 200 mg g^-1^. Table 3 illustrates the comparison of biosorption capacity of CPZiONp-composite with other biosorbents used for removal of metribuzin. This table indicates that biosorption capacity of CPZiONp-composite is comparable with other biosorbents which designates the importance of CPZiONp-composite as a biosorbent. The numerical value of average free energy E is 0.100 (kJmol^-1^) which confirms physiosorption nature of biosorption of metribuzin onto CPZiONp-composite.

**Table 2:** Coefficients of isotherm models for the biosorption of metribuzin onto CPZiONp-composite.

Isotherm	Coefficients	Values
Freundlich	K_F_ (mg g^-1^)	1.154
	1/n	0.867
	R_L_	0.61–0.87
	R^2^	0.9997
Langmuir	K_L_ (g mg^-1^ min^-1^)	0.0041
	Q_e_ (mg g^-1^)	200
	R^2^	0.9660
Temkin	a_T_ (mg g^-1^ min^-1^)	0.102
	b_T_ (kJmol^-1^)	0.113
	R^2^	0.9628
D‒R	ɛ (mol^2^ J^-2^)	5 × 10^–5^
	Q_m_ (mg g^-1^)	38.42
	E (kJmol^-1^)	0.100
	R^2^	0.8491

**Table 3:** Comparison of the biosorption capacities of different biosorbents with ZnONPs-IPPs for the biosorption of metribuzin.

Biosorbent	Biosorption capacity (mg g^-1^)	Reference
Corn cob	4.07	68
Banana peels	167	12
Wheat straw	596	69
CPZiONp-composite	200	Present study

### Kinetic studies

In the present study pseudo first-order, pseudo second-order, Elovich and Intraparticle diffusion models were applied to the biosorption data to find out the best fit kinetic model. The constants of each model were computed from the slope and intercept of their linear plots. The magnitudes of these constants are summarized in Table 4. As it seems from the value of correlation coefficient; pseudo second-order fits well to the biosorption data as compared with pseudo first-order, Elovich and Intraparticle diffusion models for the biosorption of metribuzin onto CPZiONp-composite. In addition, a favorable agreement was found between the experimental biosorption capacity (Q_e_, exp) and calculated biosorption capacity (Q_e_, cal) determined from pseudo second order in contrast to pseudo first order. The result of Intraparticle diffusion model demonstrates that experimental data does not follow the Intraparticle diffusion model because the plot of Q_t_ against t^1/2^ did not generate straight line with zero intercept.

**Table 4:** Coefficients of kinetic models for the biosorption of metribuzin onto CPZiONp-composite.

Kinetic model	Coefficients	Values
	Qe (mg g^-1^), experimental	13.251
Pseudo first-order	Q_e_ (mg g^-1^)	4.496
	K_1_ (min^-1^)	0.046
	R^2^	0.8381
Pseudo second-order	K_2_ (g mg^-1^ min^-1^)	0.021
	Q_e_ (mg g^-1^)	13.661
	R^2^	0.9987
Elovich	α (mg g^-1^ min^-1^)	5.6 × 10^4^
	β (g mg^-1^)	1.187
	R^2^	0.9217
Intraparticle diffusion	K_id_ (g mg^-1^ min^-1/2^)	0.294
	C (mg g^-1^)	10.469
	R^2^	0.9753

### Thermodynamic study

The magnitude of ΔH° was computed from the slope while magnitude of ΔS° was computed from the intercept of the plot lnK_d_ against 1/T and summarized in Table 5. A thorough assessment of the result indicates that the values of ΔG° are negative suggested that biosorption of metribuzin onto CPZiONp-composite is spontaneous process. Moreover, lower temperature is more favorable for the biosorption of metribuzin onto CPZiONp-composite because the value of ΔG° increases with rising in temperature. It has been acknowledged that ΔG° also interprets the nature of biosorption that whether the process of biosorption happen through physical or chemical. Usually, for physical biosorption the value of ΔG° is ranged from 0–20 kJol mol^-1^ while for chemical biosorption the value of ΔG° is ranged from 80–400 kJol mol^-1^^70^. The table clearly indicates that ΔG° has values in the range of 0.061 to 0.549 suggesting that biosorption of metribuzin is typically physical in nature. The table also shows that ΔH° has negative that reflects the exothermic nature of metribuzin biosorption onto CPZiONp-composite^71^. Similarly, ΔS° was found negative that demonstrates the randomness decreases at the liquid–solid interface in the course of biosorption of metribuzin onto CPZiONp-composite^72^.

**Table 5:** Thermodynamic parameters for the biosorption of metribuzin onto CPZiONp-composite.

Temperature (K)	ΔG° (kJmol^-1^)	ΔH°(kJmol^-1^)	ΔS°(kJmol^-1^ K^-1^)
303	− 0.549	− 5.326	− 0.015
313	− 0.323		
323	− 0.215		
333	− 0.061		

## Conclusion

In brief, an affective biosorbent was prepared through impregnation of zinc oxide nanoparticles on cucumber peels for the biosorption of metribuzin pesticide from aqueous media. The prepared composite was characterized by FTIR, SEM, EDX, point of zero charge and surface area pore size analyzer which indicate the synthesis of composite and variation in biosorbent nature during biosorption process. The biosorption of metribuzin onto CPZiONp-composite was depending greatly on initial solution pH, dose of CPZiONp-composite, contact time, initial metribuzin concentration and temperature. The pH study indicates the dependency of biosorption of metribuzin on the initial solution pH and maximal biosorption was obtained at pH 3.0. It is revealed from isotherm studies that Freundlich isotherm best fit the experimental data of biosorption of metribuzin onto CPZiONp-composite. The kinetic studies of the metribuzin biosorption onto CPZiONp-composite exhibit that pseudo second order furnished the perfect compatibility of the experimental data for metribuzin. The results of Gibbs free energy demonstrated the spontaneous nature and the enthalpy revealed the exothermic nature of the biosorption of metribuzin onto CPZiONp-composite. It may be concluded from the above outcomes, this composite is endorsed as an effective and low cost biosorbent for the removal of metribuzin from aqueous media. Our future research will be focused in the direction of experiments on continuous column for the removal of metribuzin from industrial effluents.
